# Management of Sjogren's Syndrome Patient: A Case Report of Prosthetic Rehabilitation with 6-Year Follow-Up

**DOI:** 10.1155/2014/761251

**Published:** 2014-11-13

**Authors:** Marcos de Mendonça Invernici, Amanda Finger Stadler, Gastão Vale Nicolau, Maria Ângela Naval Machado, Antônio Adilson Soares de Lima, Marilia Compagnoni Martins

**Affiliations:** ^1^School of Dentistry, Department of Oral Surgery and Periodontics, São Paulo University, Avenida do Café, s/n, Monte Alegre, 14040-904 Ribeirão Preto, SP, Brazil; ^2^School of Dentistry, Department of Conservative Dentistry, Federal University of Rio Grande do Sul, Rua Ramiro Barcelos 2492, 90035-003 Porto Alegre, RS, Brazil; ^3^Department of Stomatology, Federal University of Paraná, Campus Botânico, Avenida Prefeito Lothário Meissner 632, 80210-170 Curitiba, PR, Brazil

## Abstract

Completely and partially edentulous patients with Sjogren's syndrome (SS) experience severe hyposalivation, xerostomia, and considerable difficulty in using tissue-supported prosthesis. This clinical paper describes the management, treatment, and 6-year follow-up of a patient diagnosed with SS type II, who uses corticosteroids and antihyperglycemic drugs. The patient received restorative, periodontal, and surgical treatments followed by implant-supported fixed prosthesis. Radiographic evaluation and probing depth showed gingival health and no bone loss after 6 years. Treatment with implant-retained dental prosthesis greatly increased comfort and function, offering an alternative to patients with SS.

## 1. Introduction

Dental implants, as treatment in edentulous areas, have become a standard of care in the last decade. Although the literature presents many references as contraindications of this type of treatment in patients with systemic diseases, the benefits of treatment with dental implants in such patients might outweigh the risks [1].

Sjogren's syndrome (SS) is a systemic autoimmune disorder that affects the exocrine glands, particularly the salivary and lacrimal glands. The syndrome is characterized by the presence of an inflammatory infiltrate of lymphocytes interfering with the function of the glands confirmed by a biopsy of the labial gland. There are two clinical forms of SS. The primary form is characterized by dry eye conjunctiva and hyposalivation and the secondary form occurs in conjunction with other connective tissue diseases such as rheumatoid arthritis and lupus erythematosus [1, 2]. SS affects 0.5–3% of the population and is more prevalent in women than in men (9 : 1) [3]. It is usually diagnosed during the fifth decade of life but can affect all age groups [4, 5].

Oral implications of SS are hyposalivation, xerostomia, inflamed and burning oral mucosa, rampant caries, sclerosis or growth of parotid gland, frequent manifestation of erythematous candidosis, angular cheilitis, increased plaque retention, and difficulty in swallowing [6, 7]. Due to xerostomia and burning of the oral mucosa, patients with SS experience great discomfort and pain when wearing traditional removable prostheses. There is no definitive treatment for SS. Alternative therapies, such as use of lubricants and artificial saliva, increased fluid intake, and salivary stimulation, are used to relieve the symptoms. However, these treatments are palliative and the use of removable implant-retained dental prosthesis could help to relieve the discomfort in the patients.

Literature presents six case reports with SS patients treated with dental implants [8–13]. Moreover, there are few reports in the literature about clinical long-term follow-up of this type of treatment [9]. The aim of this paper is to describe a case report of a patient with SS type II that required implant-supported fixed prosthesis treatment.

## 2. Clinical Report

A 58-year-old female was referred to the Periodontology Outpatient Clinic of Federal University of Paraná Dental School, Brazil, to receive periodontal treatment and to install dental implants in areas equivalent to the superior right premolar teeth, in August 2008.

During anamnesis, the patient reported that she had been diagnosed with SS in 1989, after the appearance of some symptoms, such as xerostomia and xerophthalmia. During the same year, rheumatoid arthritis was also diagnosed. She started the use of corticosteroids prednisone, 20 mg daily, in order to control inflammation and pain. The disease has been controlled ever since. In 2006, the patient was diagnosed with type II diabetes mellitus and began the use of an oral hypoglycemic drug (sulfonylurea 80 mg) and metformin hydrochloride (500 mg) to control blood glucose. Another piece of information recorded in the preliminary case history was that the patient stopped smoking right after receiving the SS diagnosis. Besides medication, the patient related the use of eye drops and water day and night to keep her eyes and mouth moist. The report included dryness of the vaginal mucosa, hands, and feet.

**Figure 1:**
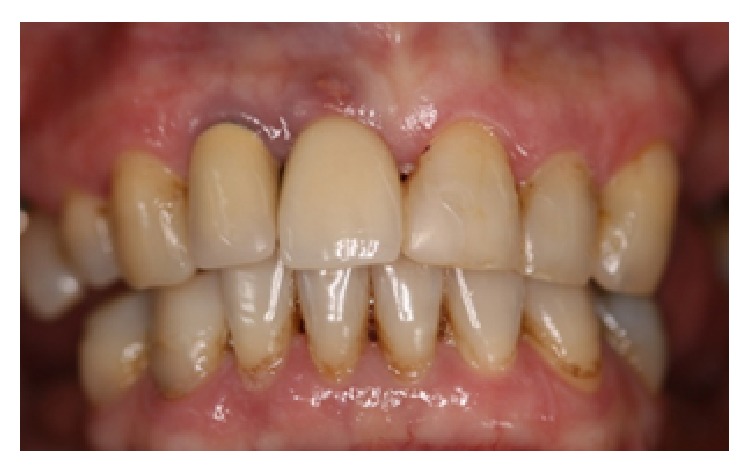
Initial clinical view of the patient with Sjogren's syndrome (August 2008).

**Figure 2:**
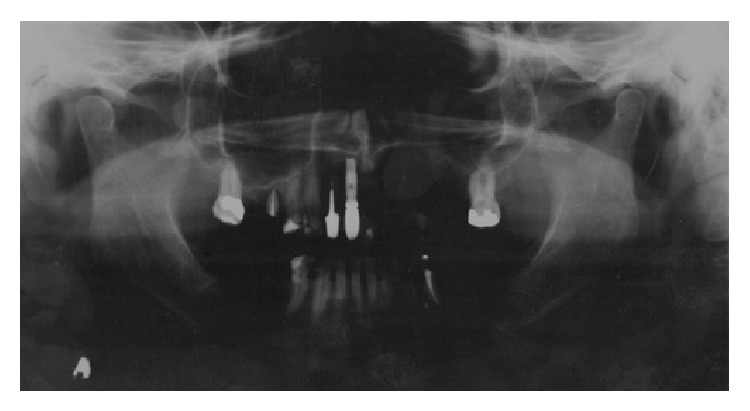
Initial panoramic radiography (August 2008).

The main complaint of the patient was lack of adaptation in using a partially removable dental prosthesis. During the intraoral examination it was observed that an implant replaced the central right incisive tooth, and several decayed teeth were noted (first quadrant: canine, first premolar, and third molar; second quadrant: canine and second molar; third quadrant: first premolar; and fourth quadrant: canine and first premolar) as well as missing teeth (first quadrant: first and second molar; second quadrant: first and second premolar and first and third molar; and third and fourth quadrants: from second premolar to third molar). Indication for endodontic treatment was observed for the lateral left upper incisive tooth and first left lower premolar tooth. The second right upper premolar had a ceramic crown with a metal core (Figure 1). The patient was also diagnosed with generalized chronic periodontitis. An initial panoramic radiograph was taken (Figure 2). Salivary flow rate was performed and the result was 0.1 mL/minute. The patient agreed to participate in the study by signing an informed consent form.

After diagnosis, a treatment plan was established to rehabilitate the patient with restorative and endodontic procedures, nonsurgical periodontal treatment, osseointegrated implants, and prosthesis. Due to the patient reporting a lack of adaptation with the removable prosthesis it was decided to try an implant-supported fixed prosthesis.

**Figure 3:**
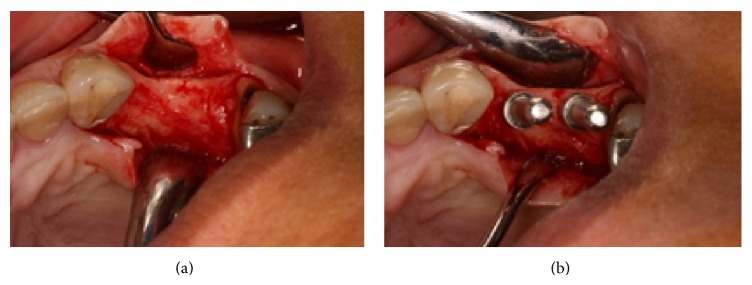
Full-thickness flap, showing the bone (a) and seating tip in position (b) (November 2008).

In October 2008 the patient was submitted to a nonsurgical periodontal treatment at the periodontology outpatient clinic. One month later, two titanium implants (Systhex Sistema de Implantes Osseointegrados Ltda., Curitiba, Brazil) were placed in the alveolar ridge corresponding to the left upper region of the first and second premolar with the following specifications: internal hexagon, platform 4.3 mm, first implant 4.0 × 10 mm, and second implant 4.1 × 11.5 mm. Both implants had acid-attached surfaces. A full-thickness mucoperiosteal flap was elevated bilaterally, in order to access the bone (Figure 3(a)) and seat the tip in position (Figure 3(b)). The bone density of the patient was type II [14]. The flaps were repositioned and silk sutures (4.0 Ethicon, Inc., Somerville, NJ, USA) were used for closure. The patient was premedicated 1 hour before surgery with amoxicillin 1 g (Infabra Ind. Farm. Bras. Ltda., Rio de Janeiro, Brazil).

Postoperatively, an antibiotic was prescribed (three times a day, for 1 week; Amoxicillin, 500 mg, Infabra Ind. Farm. Bras. Ltda., Rio de Janeiro, Brazil) as well as an analgesic (four times a day for 48 hours, Paracetamol, 750 mg, Medley Indústria Farmacêutica Ltda., Campinas, Brazil). There was no need to prescribe a corticosteroid anti-inflammatory drug for edema control, since the patient already used this on a daily basis. An antiseptic mouthwash with chlorhexidine gluconate 0.12% (Colgate-Palmolive Company, New York, NY, USA) was used twice daily for 1 week. Sutures were removed after 7 days.

The patient was referred to Endodontics and Restorative Dentistry Outpatient Clinics for other needs.

**Figure 4:**
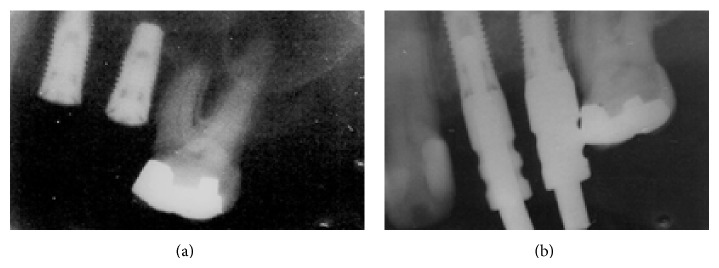
Periapical radiograph four months after surgery (a) and connection of the abutments (b) (March 2009).

After four months, the patient returned and the surgical site was reopened in order to install abutments (4.0 mm). A clinical exam to evaluate the implant's osseointegration was performed and implants showed no mobility. Periapical radiographs were taken (Figure 4(a)). One week later, an impression for a prosthesis was carried out and a new periapical radiography was taken to check the abutment connection (Figure 4(b)). The final prosthesis was installed three weeks later.

**Figure 5:**
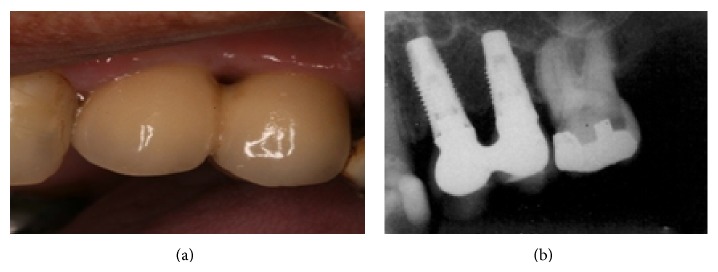
Six-year follow-up: clinical view of the implant-supported fixed prosthesis (a) and periapical radiograph of implants showing no bone loss (b) (May 2014).

**Figure 6:**
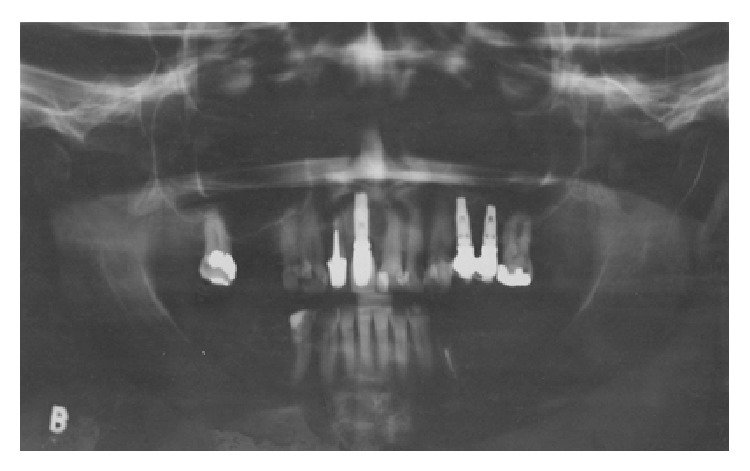
Final panoramic radiography (May 2014).

The patient was followed up every 3 months with a clinical exam, and after a 6-year follow-up, periodontal status and implants were accessed during clinical examination (Figure 5(a)) with periapical (Figure 5(b)) and panoramic radiographs (Figure 6). The probing depth in all sites had less than 3 mm without bleeding, and the radiography showed no bone loss; if implants had biofilm or bleeding on probing, a periodontist performed prophylaxis. The implant-supported fixed prosthesis remained in function.

The patient still needs rehabilitation with implants in the upper right region and bimandibular lower regions. This rehabilitation has not been performed yet due the patient's concern about cost.

## 3. Discussion

Results of this clinical report, with a 6-year follow-up, showed that this patient diagnosed with Sjogren's syndrome and rheumatoid arthritis could be treated successfully with dental implants and a fixed prosthesis. This is in accordance with available literature [4, 11, 12, 15]. The patient required treatment with corticosteroids, which may change the osseointegration process [16] and may result in bone loss around the implants [11]. However, during follow-up, no complications were diagnosed in the repair process, and the level of crestal bone around implants remained unchanged. This situation differs from that reported by Payne et al. [11], in which a patient with SS treated with cortisone therapy showed bone loss around the implants.

The patient also had a diagnosis of diabetes mellitus, and the literature shows that there is a significant correlation between oral dryness and type II diabetes mellitus [17]. A recent systematic review found a difference in statistics between diabetic and nondiabetic patients, with regard to marginal bone loss, with less bone loss for the nondiabetic patients [18]. Nevertheless, this patient showed no bone loss during the 6-year follow-up, despite the diagnosis of Sjogren's syndrome, rheumatoid arthritis, and diabetes mellitus.

Literature shows that patients with SS have difficulties with eating, swallowing, and speaking and present a high prevalence of caries. A recent publication also found a significant correlation between oral dryness and the quality of sleep, anxiety, and depression [17]. Usually, these patients report regular dental visits over a long period of time and an increased number of decayed teeth between visits, in spite of good oral hygiene [2]. Tooth decay is accelerated as a result of a reduction in salivary flow rate and the subsequent loss of saliva antibacterial properties in a dry mouth. In fact, unexplained rampant dental caries may be the first sign of a dry mouth. The factors related to pathogenesis of dental caries in SS may be the loss of the salivary buffering capacity and the increased intake of cariogenic drinks [19]. Even with excellent levels of oral hygiene, tooth decay and premature loss are frequent in SS patients. It is not unusual for patients with a more advanced disease to be edentulous or to have complete dentures [6, 8]. They also have a higher DMFT (decayed/missing/filled teeth) score compared to individuals in the control group [19]. In the present case report, the patient presented at the initial exam showing several missing and decayed teeth. Severe caries activity might be the cause of early tooth loss.

Although the literature presents 6 papers [8–13] concerning SS patients treated with dental implants, no systematic investigations have yet been done to evaluate the influence of SS and its effect on bone healing and remodeling after implant placement [11]. Minimal long-term clinical documentations also exist in literature regarding this type of treatment [9].

Often the rehabilitation of patients with SS is neglected and only symptomatic treatment is performed. As a result of continuous hyposalivation, patients with SS consequently have various dental treatments that fail. This explains why it is so complex to finish the research subject's oral rehabilitation and the reason this patient remains in treatment. Management of SS requires multidisciplinary dental care, mainly restorative, endodontic, prosthetic, and implant rehabilitation. After care and rehabilitation it is necessary to maintain continuous follow-up over a long period of time.

One reason motivating the treatment of patients with SS with dental implants is that they have difficulty in adapting to removable partial dentures, due to hyposalivation and, in some cases, the report of the sensation of a burning mouth. There are no published articles about the immediate prosthetic loading in patients with SS. This was the main reason why the authors decided to use a 2-stage implant treatment, with prosthetic loading after 5 months.

## 4. Conclusion

Within the limitations of this case report, we can suggest that it is possible to treat partial edentulous patients with Sjogren's syndrome associated with rheumatoid arthritis and diabetes, using corticosteroids and oral hyperglycemic therapy with dental implants and fixed prosthesis. This treatment improved the patient's comfort and showed no bone loss after 6 years. Therefore, more studies are needed to validate the effects of the implant therapy and fixed prosthesis in patients with Sjogren's syndrome.
